# Dynamics of Adaptive Immune Cell and NK Cell Subsets in Patients With Ankylosing Spondylitis After IL-17A Inhibition by Secukinumab

**DOI:** 10.3389/fphar.2021.738316

**Published:** 2021-10-14

**Authors:** Yutong Jiang, Mingcan Yang, Yanli Zhang, Yefei Huang, Jialing Wu, Ya Xie, Qiujing Wei, Zetao Liao, Jieruo Gu

**Affiliations:** Department of Rheumatology and Immunology, Third Affiliated Hospital of Sun Yat-sen University, Guangzhou, China

**Keywords:** ankylosing spondylitis, T cell, B cell, IL-17A, Th17 cell

## Abstract

**Background:** Anti-IL-17A therapy is generally effectively applied in patients with Ankylosing Spondylitis (AS) to achieve and maintain remission. However, the influence of anti-IL-17A on the composition of the immune system is not apparent. Our prospective study was to explore the changes in immune imbalance regarding T cell, B cell and natural killer (NK) cell subsets after secukinumab treatment in AS patients.

**Methods:** Immune cell distribution of 43 AS patients treated with secukinumab for 12 weeks and 47 healthy controls (HC) were evaluated. Flow cytometry using monoclonal antibodies against 25 surface markers was accomplished to explore the frequencies of lineage subsets. The differences between HC, AS pre-treatment, and post-treatment were compared using the paired Wilcoxon test, Mann-Whitney *U* test, and ANOVA.

**Results:** AS patients had altered immune cell distribution regarding T cell and B cell subsets. Apart from activated differentiation of CD4^+^ T cell, CD8^+^ T cell and B cell, higher levels of cytotoxic T (Tc) two cells and Tc17 cells were noted in AS patients. We confirmed that helper T (Th) one cell became decreased; however, Th17 cells and T follicular helper (Tfh) 17 cells went increased in AS. After 12 weeks of secukinumab therapy, CRP and ASDAS became significantly decreased, and meanwhile, the proportions of Th1 cells, Tfh17 cells and classic switched B cells were changed towards those of HC. A decreased CRP was positively correlated with a decrease in the frequency of naïve CD8^+^ T cells (*p =* 0.039) and B cells (*p =* 0.007) after secukinumab treatment. An elevated level of T cells at baseline was detected in patients who had a good response to secukinumab (*p* = 0.005).

**Conclusion:** Our study confirmed that AS patients had significant multiple immune cell dysregulation. Anti-IL-17A therapy (Secukinumab) could reverse partial immune cell imbalance.

## Background

Ankylosing Spondylitis (AS) is a chronic inflammatory disease with complex etiology. Other than the genetic contribution of HLA-27 and other genes (Fiorillo et al., 2019), the innate and the adaptive immune system, driven primarily by Th1 and Th17 cells, contribute to pathogenic processes of AS. Human genetics and animal model studies strongly support the notion that the cytokines tumor necrosis factor (TNF)-α and interleukin (IL)-17 exert a substantial influence on AS pathogenesis (Sieper and Poddubnyy, 2017; Watad et al., 2018). TNF-α inhibitor (TNFi) has become a widely used medication for AS. According to the ASAS/EULAR recommendations, biologic DMARD therapy, typically TNFi therapy, should be considered in patients with persistently high disease activity despite conventional treatments (Noureldin and Barkham, 2018). We have reported AS patients have altered immune cell frequencies, including CD4^+^ T cells, CD8^+^ T cells and B cell, and found that anti-TNF-α therapy could improve the frequency of immune dysregulation of CD4^+^ T cells and negative regulatory cells (Yang et al., 2020). Although most patients had improvement with TNFi therapy, there is an unmet need that not all patients respond well to or can tolerate TNFi treatment (Noureldin and Barkham, 2018).

IL-17, produced predominately by CD4^+^ T helper (Th) 17 cells, is also a significant proinflammatory cytokine linked to pathogenic processes in autoimmune and inflammatory diseases, especially in AS. Blockade of IL-17 has become a promising treatment option (Sieper and Poddubnyy, 2017). As a fully human monoclonal antibody against IL-17A, secukinumab (Cosentyx^®^) received FDA approval to treat AS and psoriasis arthritis (PsA) in 2016. Sustained, long-time efficacy of secukinumab in patients with AS have also been reported (Pavelka et al., 2017; Baraliakos et al., 2018). However, these clinical trials revealed that some patients did not respond well to anti-IL-17A therapy, the reason of which remains unclear. Given side-effects and expenses of biological agents, it is required to identify biomarkers to predict treatment effects.

Limited literature explored the function of natural killer (NK) cells in AS. RNA sequencing of blood cells from AS patients and controls identified downregulated genes were enriched in CD8^+^ T cells and NK cells, revealing NK cells might take part in the pathogenesis of AS (Li et al., 2017).

Downstream effects of IL-17 blockade are worthy of exploration for a better understanding of this biological agent. Here, we conducted a prospective study 1) to verify the immune imbalance in AS patients; 2) to analyze clinical improvement and the alteration in immune cell frequency after IL-17A inhibition by secukinumab; and 3) to explore the predictors of good responses to secukinumab in AS patients.

## Materials and Methods

### Study Population

A prospective study was designed to observe lymphocyte alteration and short-term efficacy after 12 weeks of secukinumab treatment in patients with AS. We included AS patients from Department of Rheumatology and Immunology at Third Affiliated Hospital of Sun Yat-sen University. All the patients fulfilled 1984 Modified New York Criteria (van der Linden et al., 1984) and had high disease activity (ASDAS ≥ 1.3). Patients with severe infection, had a vaccination, biologic agents, unstable use of non-steroid anti-inflammatory drugs (NSAIDs) or disease modifying anti-rheumatic drugs (DMARDs) within 3 months prior to the study and pregnant women were excluded from the study. Demographic and clinical variables, including age, sex, disease duration, clinical manifestation, medication, C-reactive protein (CRP) and ASDAS were recorded at baseline and after secukinumab treatment. All the patients received a subcutaneous injection of secukinumab (Cosentyx^®^) 150 mg at week 0, 1, 2, 3, 4, 8, and 12. Physician’s overall assessment (good response, no response) was acquired to evaluate the treatment effect of secukinumab.

Healthy controls (HC) were recruited from healthy volunteers at our hospital. HC with diagnosed chronic diseases, a complaint of back pain, skin rash, and other possible symptoms of AS, medication intake and positive family history of AS were excluded from the study. Blood samples (Heparin sodium tube 5 ml and blood collection tube without anticoagulant 3 ml) were acquired from the participants. The entire study was conducted from March 2020 to February 2021. All the participants gave written consent forms.

### Flow Cytometry

Peripheral blood mononuclear cells (PBMCs) were acquired and then were stained for surface markers for 20 min in PBS containing fluorescent antibodies. Fluorescently labeled antibodies included CD3-PerCP-Cy5.5, CD25-PE, CD45RA-FITC, CD8-PerCP-Cy5.5, CD19-PerCP-Cy5.5 and CD56-PE-Cy7 (Tianjin Three arrows, China); CD4-APC-H7, CD8-BV510, CD127-BV421, CCR7-AF647, CD28-PE-Cy7, CD3-APC-H7, CD4-PE-Cy7, CXCR3-Alexa488, CXCR6-BV510, CXCR5-Alexa647, CCR4- BV421, PD-1-PE, CD45-APC-H7, CD27-BV421, IgD-BB515, IgM-BV510, CD38-APC, CD24-PE, and CD21-PE-Cy7 (BD, United States). The instrument settings and gating strategies were adopted from previous works (Supplementary Figure S1) (Yang et al., 2020; Zhu et al., 2020). All experiments, including cell separation and sample preparation, were performed according to standardized experimental manuals. Samples were analyzed using CytoFLEX flow cytometer (Beckman, United States). Results are expressed as the proportion of cells expressing particular markers. T cell subsets, including cytotoxic T (Tc) cells, helper T (Th) cells, T follicular helper (Tfh) cells, regulatory T (Treg) cells, B cells and NK subsets were identified (Supplementary Table S1).

### Statistical Analyses

First, we performed a descriptive analysis of the participants. Data with normal distribution were stated as mean ± standard deviation, and those with non-normal distribution were recorded as median (interquartile range). Comparisons between subgroups were completed using paired Wilcoxon test, Mann-Whitney *U* test, and ANOVA. We applied the Pearson correlation or Spearman’s rank correlation analysis to examine the relationship between clinical parameters and the frequency of immune cell subtypes. Cluster analyses of immunophenotypic variables were performed using R package. A *p* value less than 0.05 was considered significance. All the analyses were completed using SPSS, release 20.0 (IBM, Armonk, NY, United States), and graphs were made using Prism (GraphPad Software, Inc., La Jolla, CA, United States).

## Results

### General Characteristics of the Participants

Totally 45 AS patients and 47 HC were included in the current study. Two patients who did not receive secukinumab at the specific time-point were excluded for further analysis of immune cell frequency. The mean age of 43 patients was 28.3 ± 9.9 years in AS group. The median disease duration was 6.5 (3.1–9.6) years. The median C-reactive protein (CRP) was 17.7 (1.5–23.8) mg/L at baseline (Supplementary Table S2). Age- and sex-matched HC had a mean age of 30.6 ± 5.1 years.

### Changes in T Cell Subsets Between Ankylosing Spondylitis and Healthy Controls

**FIGURE 1:**
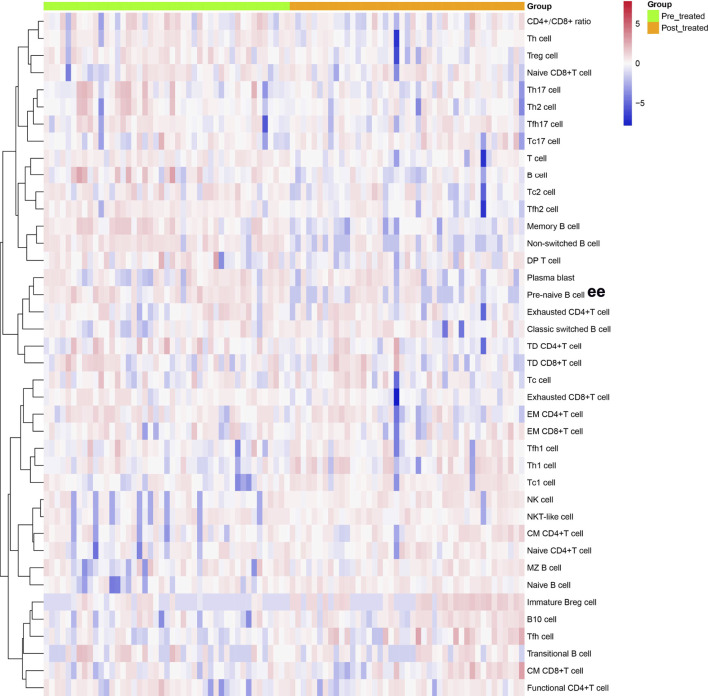
Cluster analyses of immune cell frequency in the patients with AS before and after treated with secukinumab. Each column represented individual patients with AS. Pre-treatment group was marked as green and post-treatment group was marked as orange. The rows represented immune cell subsets that are differentially expressed. The magnitude of parameter expression was color-coded with red for an increase in expression and blue for a decrease in expression. Th cell, helper T cell; Treg cell, regulatory T cell; Tc cell, cytotoxic T cell; Tfh cell, follicular helper T cell; DP T cell, double positive T cell; TD, terminally differentiated; EM, effector memory; CM, central memory; NK cell, natural killer cell; Breg cell, regulatory B cell.

**FIGURE 2:**
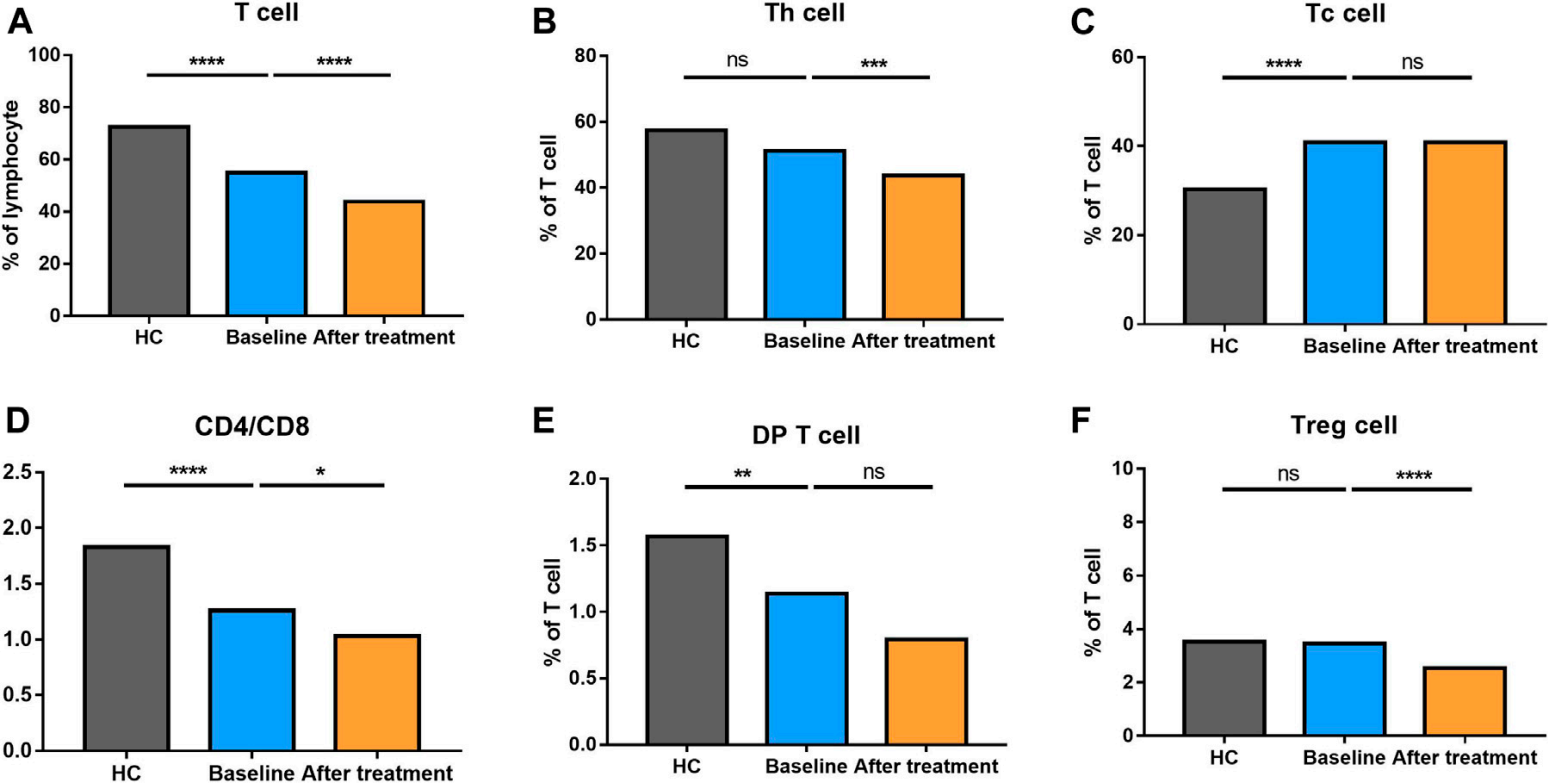
Altered expression of T cell subsets in AS patients after treated with secukinumab. The proportion of T cell subsets measured by flow cytometry at baseline and after receiving secukinumab in AS patients. ns, not significant; *, *p* < 0.05; **, *p* < 0.01; ***, *p* ≤ 0.001; ****, *p* < 0.0001.

**FIGURE 3:**
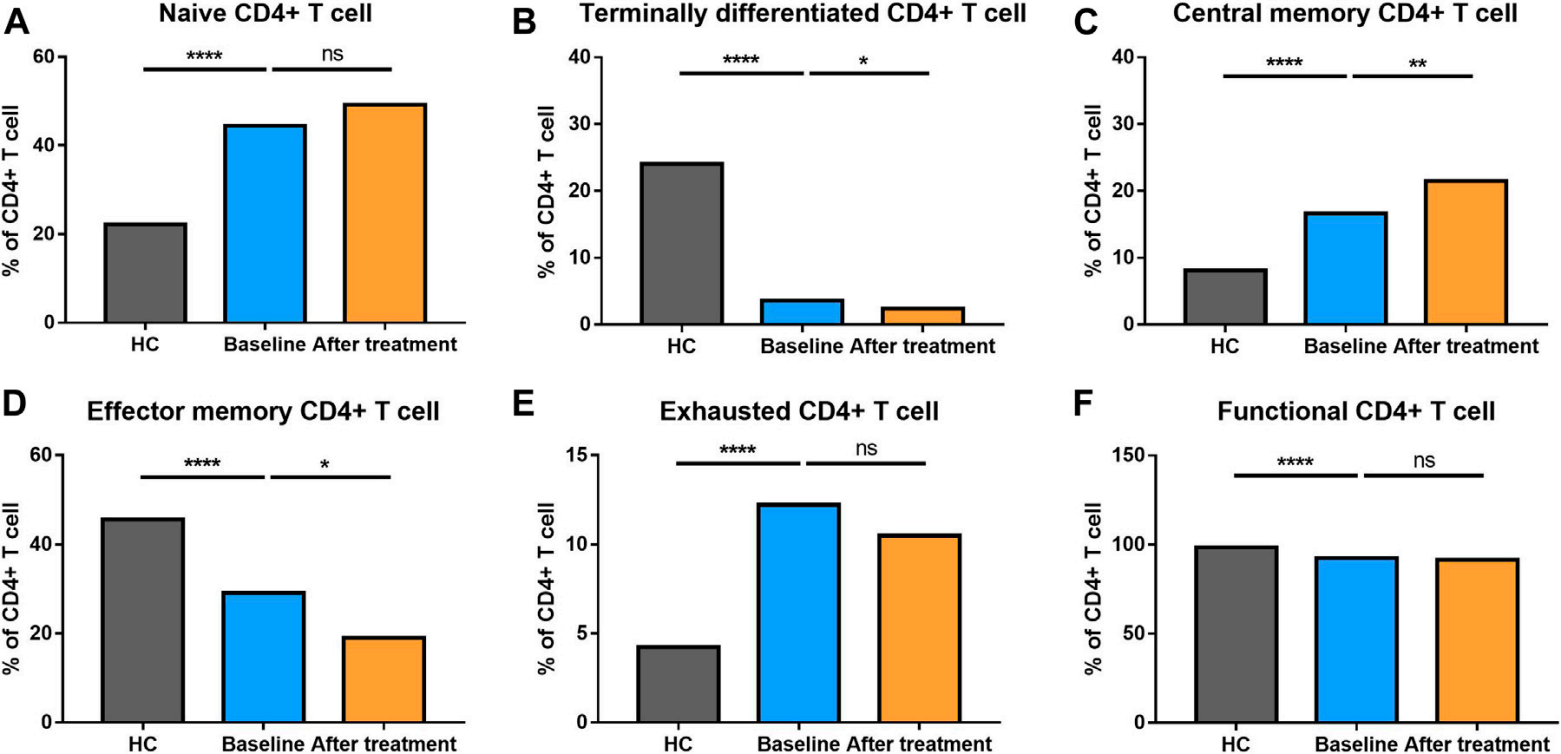
Altered expression of CD4^+^ T cell subsets in AS patients after treated with secukinumab. The proportion of CD4^+^ T cell subsets measured by flow cytometry at baseline and after receiving secukinumab in AS patients. ns, not significant; *, *p* < 0.05; **, *p* < 0.01; ***, *p* ≤ 0.001; ****, *p* < 0.0001*.*

We analyzed different differentiation stages of immune cells and found significant changes between AS patients and HC (Figure 1). AS patients had a lower proportion of total T cells, CD4/CD8 ratio, and double-positive (DP) T cells (Figure 2). In CD4^+^ T cell subsets, the proportions of naïve CD4^+^ T cells, central memory CD4^+^ T cells and exhausted CD4^+^ T cells became significantly increased, but the levels of terminally differentiated CD4^+^ T cells, together with effector memory CD4^+^ T cells, were reduced significantly in AS (Figure 3).

**FIGURE 4:**
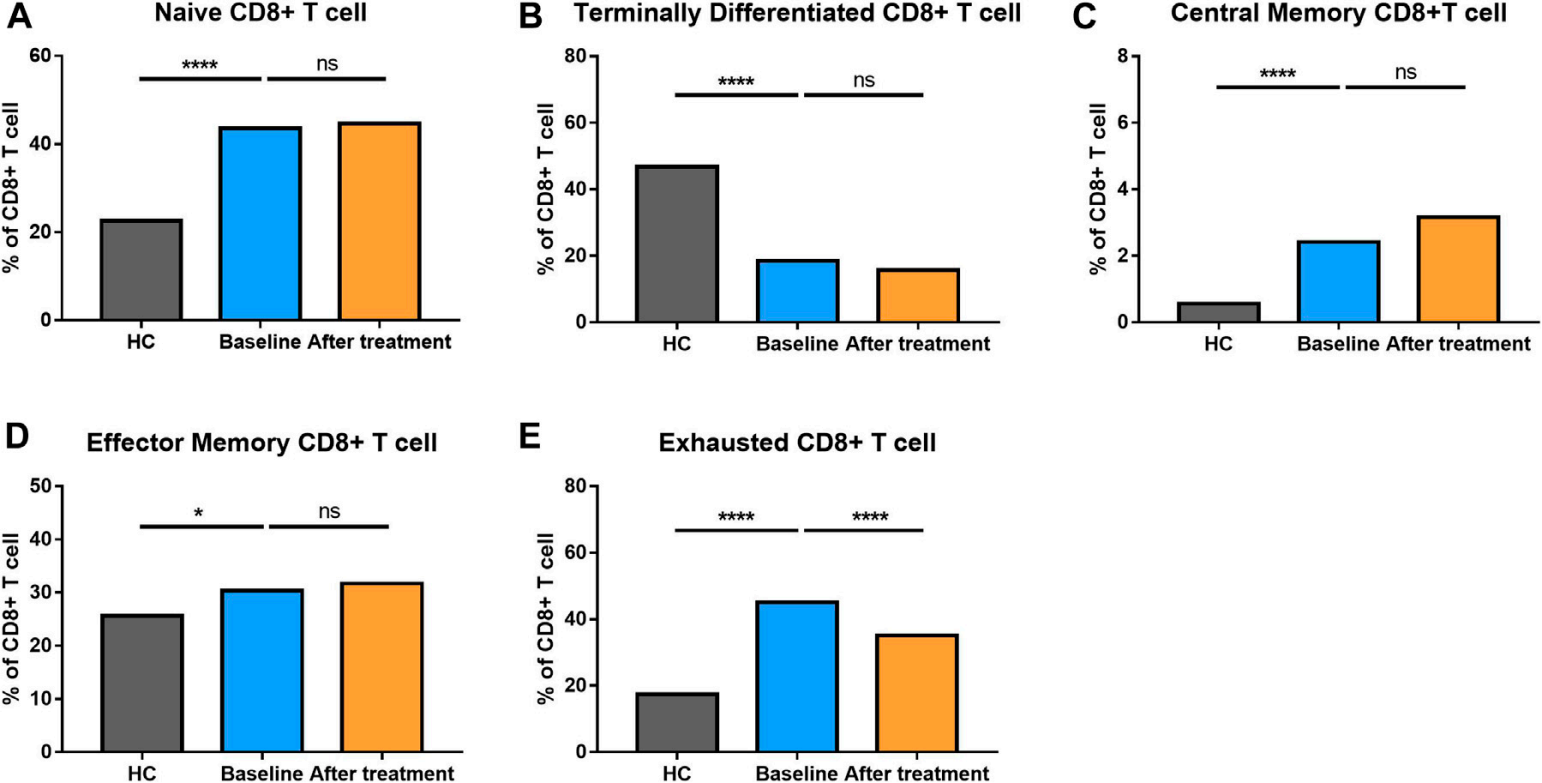
Altered expression of CD8^+^ T cell subsets in AS patients after treated with secukinumab. The proportion of CD8^+^ T cell subsets measured by flow cytometry at baseline and after receiving secukinumab in AS patients. ns, not significant; *, *p* < 0.05; **, *p* < 0.01; ***, *p* ≤ 0.001; ****, *p* < 0.0001.

The proportion of CD8^+^ T cells at different stages of differentiation was also determined (Figure 4). AS patients had elevated levels of naïve CD8^+^ T cells, central memory CD8^+^ T cells, effector memory CD8^+^ T cells and exhausted CD8^+^ T cells, but comparatively, a reduced level of terminally differentiated CD8^+^ T cells.

**FIGURE 5:**
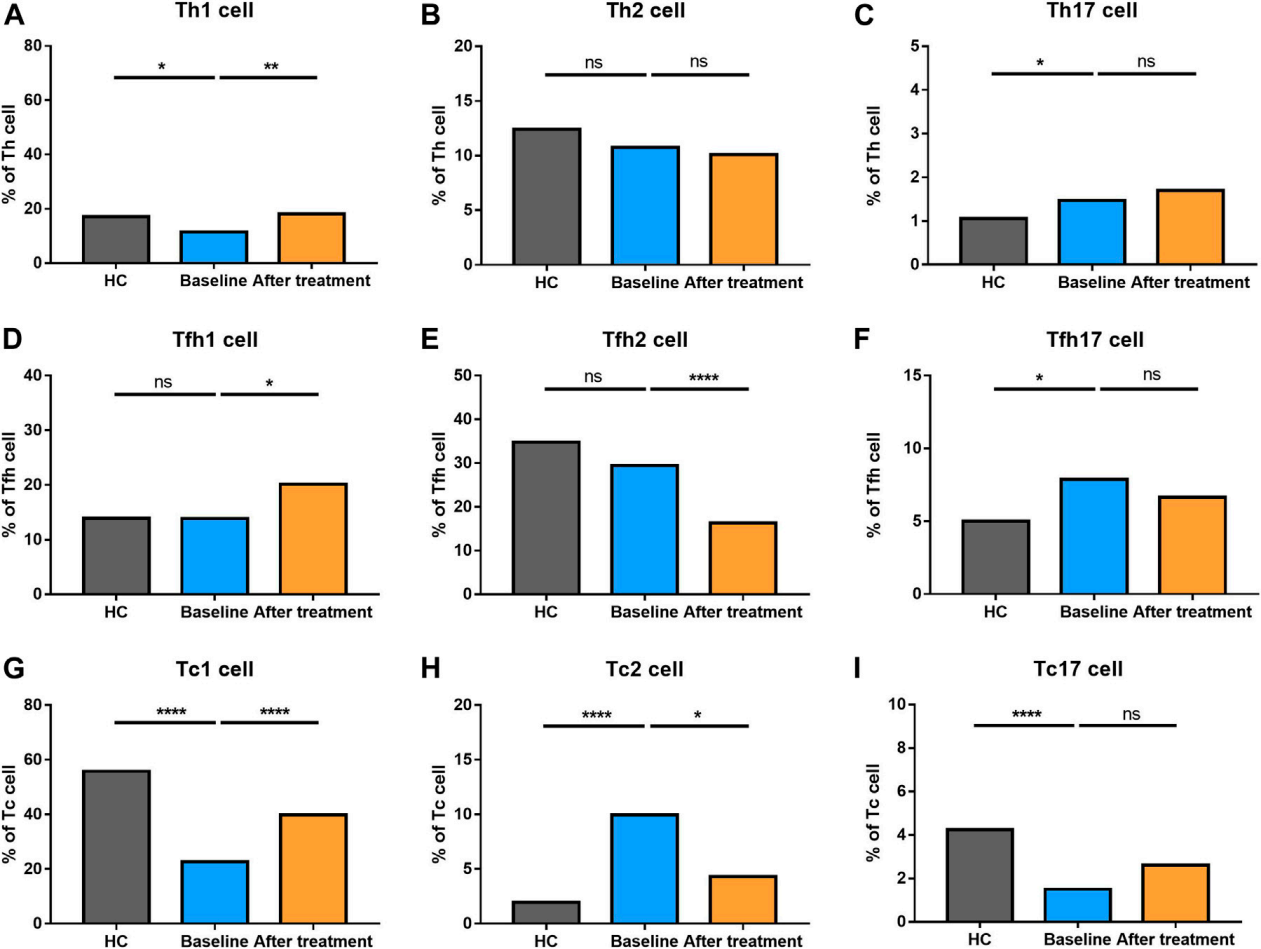
Altered expression of Th, Tfh, and Tc cell subsets in AS patients after treated with secukinumab. The proportion of Th, Tfh, and Tc subsets measured by flow cytometry at baseline and after receiving secukinumab in AS patients. Th cell, helper T cell; Tfh cell, follicular helper T cell; Tc cell, cytotoxic T cell; ns, not significant; *, *p* < 0.05; **, *p* < 0.01; ***, *p* ≤ 0.001; ****, *p* < 0.0001.

We also explored the proportion of Th (CD3^+^CD4^+^) cell subsets (Th1 cells, Th2 cells and Th17 cells), Tfh (CD3^+^CD4^+^CXCR5^+^) cell subsets (Tfh1 cells, Tfh2 cells, Tfh17 cells), and Tc (CD3^+^CD8^+^) cell subsets (Tc1 cells, Tc2 cells, Tc17 cells) between AS and HC (Figure 5). There were no significant changes in total Th cells and Tfh cells between the two groups. However, there was a significantly higher proportion of Tc cells in AS patients, together with a higher level of Tc2 cells. Comparatively, lower levels of Tc1and Tc17 cells were found in AS group. Th1 cells became decreased; however, Th17 cells went up in AS patients. The proportion of Tfh17 cells were increased in the patients’ group, while there were no differences in the proportion of Th2 cells, Tfh1 cells and Tfh2 cells. The results reflected that AS patients had a significantly altered proportion of T cell subsets.

### Changes in B Cell Subsets Between Ankylosing Spondylitis and Healthy Controls

**FIGURE 6:**
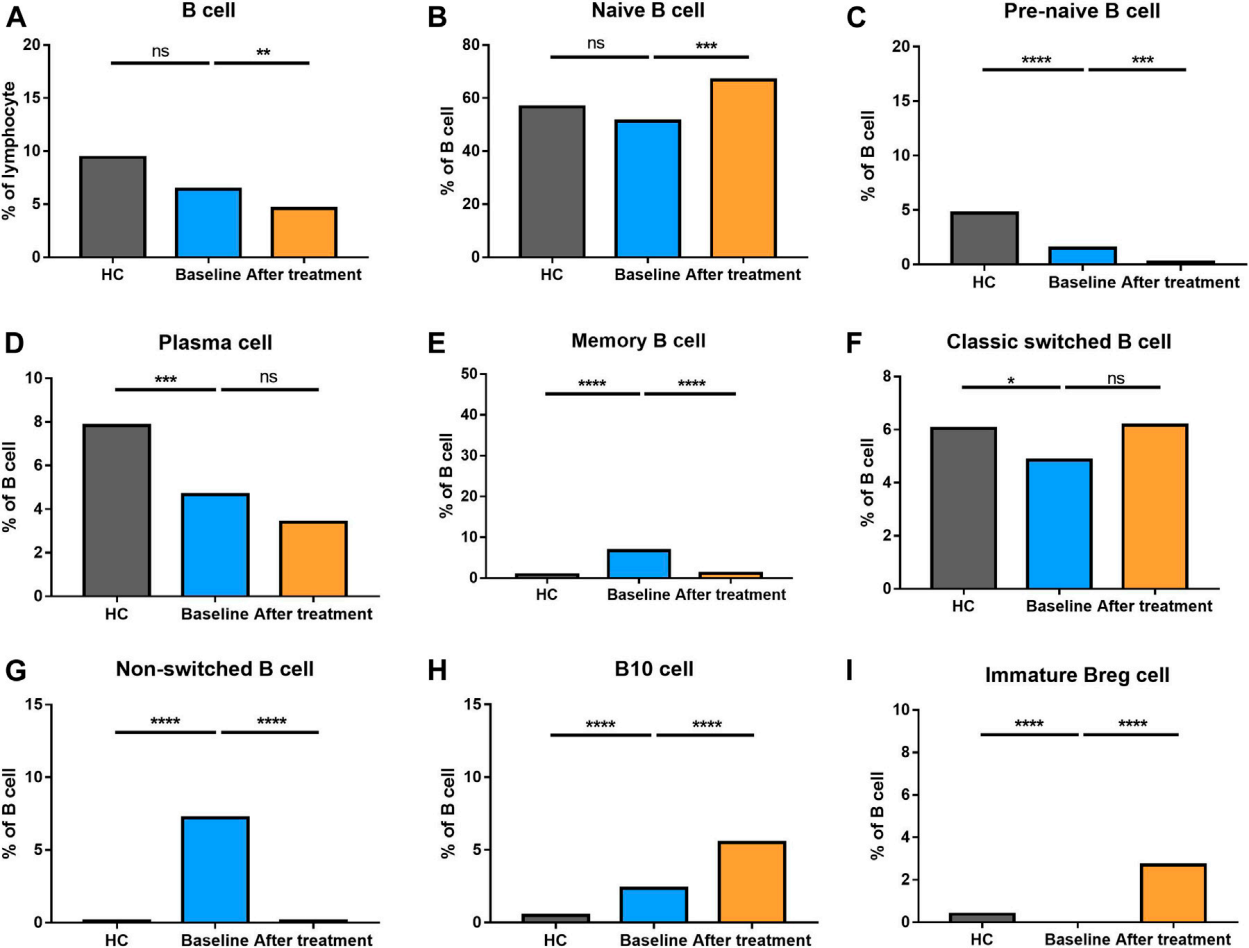
Altered expression of B cell subsets in AS patients after treated with secukinumab. The proportion of B cell subsets measured by flow cytometry at baseline and after receiving secukinumab in AS patients. Breg, regulatory B cell. ns, not significant; *, *p* < 0.05; **, *p* < 0.01; ***, *p* ≤ 0.001; ****, *p* < 0.0001.

The percentage of B cells at different stages of differentiation was compared in Figure 6. In B cell subtypes, the proportions of B cells, naïve B cells, marginal zone (MZ) B cells, and transitional B cells were not altered in AS group. However, pre-naive B cell, plasma cells, and classic switched B cells were decreased. Nevertheless, memory B cells, non-switched B cells and B10 cells became increased in AS patients compared to HC.

### Changes in Regulatory Lymphocytes Between Ankylosing Spondylitis and Healthy Controls

As shown in Figures 1, 5, regulatory lymphocytes including Treg cells and Breg cells were evaluated. We found that Breg cells were significantly lower in AS. Treg also became decreased in AS patients; however, no significant difference was detected between the groups (*p* > 0.05).

### Altered Distribution of T Cell Subsets After Secukinumab Therapy

None of AS patients underwent safety issues during the treatment or quitted the study for personal reasons. After 12 weeks of secukinumab therapy, CRP and ASDAS became significantly decreased (Supplementary Table S3). There were significant differences in immune cell frequency at baseline and after treatment (Figures 1–6). Both T cells and B cells were decreased after treated with secukinumab. The proportion of CD4^+^ T cells was reduced, leading to a lower CD4/CD8 ratio. In CD4^+^ T cell subsets, central memory CD4^+^ T cells were increased, while terminally differentiated CD4^+^ T cells and effector memory CD4^+^ T cells became decreased after anti-IL-17A therapy.

Th2 cells, Th17 cells and Tc17 cells remained stable after IL-17A inhibition. Tfh1 cells turned elevated while Tfh2 cells became reduced after secukinumab treatment. An increased level of Th1 cells and Tc1 cells, together with a decreased number of Tfh17 cells and Tc2 cells, were noticed after anti-IL-17A therapy. Moreover, the levels of Th1 cells and Tfh17 cells were changed towards those of HC after secukinumab treatment.

### Altered Distribution of B Cell Subsets After Secukinumab Therapy

Whereas the total number of B cells were reduced, the proportion of naïve B cells and classic switched B cells became elevated after anti-IL-17A therapy. Comparatively, the proportion of pre-naïve B cells, memory B cells and non-switched B cells went down significantly. Plasma cells did not alter after the treatment. These results revealed a complex alteration of T cell and B cell subsets in AS patients who underwent secukinumab treatment.

### Altered Distribution of Regulatory Cells, NK Cells and NKT-Like Cells After Secukinumab Therapy

Both the circulating regulatory T cells and B cells were changed significantly after secukinumab treatment. Treg cells were reduced, while Breg cells were increased after anti-IL-17A treatment. We also tested the proportions of NK cells (CD3^−^CD56^+^) and NKT-like cells (CD3^+^CD56^+^) and found that NKT-like cells were significantly reduced, while NK cells remain stable after treatment with secukinumab in AS patients.

### Correlation Between Clinical Variables and Immune Cell Frequency

To explore the correlation between changes of CRP values and variations in immune cell frequency after secukinumab therapy, we performed Spearman’s rank correlation analyses and found that the decrease in CRP was positively correlated with the decrease in the frequency of naïve CD8^+^ T cells (rho = 0.331, *p =* 0.039) and B cells (rho = 0.423, *p =* 0.007).

To further explore the predictors of good responses of secukinumab, we compared immune cell frequency between the subgroups who had a great response to secukinumab (*n* = 33) and those who failed to have satisfying improvement (*n* = 10) according to physicians’ overall assessment. There was no difference in age, CRP and ASDAS between the two subgroups. The only difference was an elevated level of T cells at baseline in those secukinumab responders (60.4 (51.9–66.4)) compared to non-responders (36.4 (25.5–55.7)) (*p* = 0.005).

## Discussion

Apart from the contribution of the HLA-B27 and the interaction with Tc cells, the differentiation of CD4^+^ T cells and the cytokines secreted are proved to participate in the pathogenesis of AS. Recent studies have highlighted the role of Th17/Treg imbalance and IL-17A/IL-23 cytokine dysregulation in AS (Lee, 2018). Anti-IL-17A (secukinumab) is recommended in active AS patients according to previous guidelines (Noureldin and Barkham, 2018). How this biological agent regulates immune cells remains unclear. Our study first explored the proportions of various subsets of peripheral T cells and B cells, together with NK cells, after anti-IL-17A treatment, and described that anti-IL-17A treatment could lead to the alteration of immune cells, which may be related to therapeutic functions. In these lymphocytes, Th1 cells, Tfh17 cells and classic switched B cells were changed towards those of HC after secukinumab treatment. Moreover, the baseline level of T cells might be an indicator of the excellent treatment effect of secukinumab.

Recent studies have reported immune cell dysregulation related to inflammatory disorders play a significant role in the pathogenesis of AS (Fiorillo et al., 2019). We proved again that AS patients have altered CD4^+^ T cell and CD8^+^ T cell subsets at different stages of differentiation, revealing that both CD4^+^ T cells and CD8^+^ T cells were activated in disease processes (Yang et al., 2020). CD4^+^ T cell subgroups can transform to each other and play diverse roles under appropriate environments. Naïve CD4^+^ T cells differentiate into Treg cells under the induction of TGF-β and differentiate into Th17 cells under the combined action of TGF-β and IL-6 or IL-21. Th17 cells produce cytokines including IL-17A, IL-17F, IL-21 and IL-22, thus participating in the inflammatory response, while Treg cells play a negative immunomodulatory role after being activated by homologous antigens. Th17 and Treg cells can undergo phenotypic transformation under certain conditions. In recent years, immunopathological studies on autoimmune diseases and inflammatory diseases have found that Th17/Treg imbalance is an essential factor in the occurrence and development of such diseases (Miossec and Kolls, 2012). Our findings also proved that Th1 cells became decreased, while Th17 cells were increased with a disturbed Th17/Treg balance in AS patients, which was consistent with previous reports (Xueyi et al., 2013; Fasching et al., 2017; Hajialilo et al., 2019).

IL-17A, expressed by Th17 cells and multiple lineages of the innate immune system, can act as a chemokine directly on immune cells, thus bridging innate and adaptive immunity (Gaffen, 2009; Miossec and Kolls, 2012). The inhibition of IL-17A would exert various physiological effects more than the suppression of Th17 cell activity (Patel et al., 2013). As a highly selective, fully human immunoglobulin G1k (IgG1k) monoclonal antibody directed against the IL-17A cytokine, secukinumab has been assessed in the treatment of some autoimmune diseases (Patel et al., 2013). Anti-IL-17A has become a second-line therapy for treating AS according to international guidelines (Kiltz et al., 2012). Like other biologicals, agents targeting IL-17A have the theoretical potentials to influence the immune system (Patel et al., 2013). Our study also demonstrated that secukinumab could lead to multiple variations of innate and adaptive immune responses. Th1 cells went back to normal after 12 weeks’ secukinumab treatment, and meanwhile, Th17 cells and Th17/Treg were also reduced but still higher than healthy participants. The total of CD8^+^ T cells remained stable; however, the proportion of Tc1, Tc2 and Tc17 cells were changed toward those of HC. A more extended observation is needed to explore when Th17 cells and Th17/Treg could reduce to a normal range after secukinumab treatment.

As is known, Tfh cells can support effector B cells and boost autoimmunity. Tfh17 cells help induce naïve B cells to produce immunoglobulins *via* IL-21, which is essential for B cell proliferation and differentiation (Bautista-Caro et al., 2014). The presence of antibodies targeting anti-CD74 autoantibodies (Riechers et al., 2019), protein phosphatase magnesium-dependent 1A (Kim et al., 2014), a variety of microbial components (Wen et al., 2017) in patients with AS are suggestive of B cells’ contribution. We also found that the proportion of Tfh17 cells and classic switched B cells was increased significantly, indicating B cells differentiation may also take part in the pathogenesis of AS. After IL-17A inhibition, the levels of Tfh17 cells and classic switched B cells were changed towards those of HC, reflecting the effect of anti-IL-17A therapy on B cell differentiation. Noticeably, total B cells were decreased significantly after secukinumab treatment, which was opposed to the changes brought by anti-TNF therapy (Lin et al., 2009; Yang et al., 2020).

NK cells play a vital role in innate immune responses. CD56^+^ T cells are a set of pro-inflammatory lymphocytes with some characteristics of NK cells (Krijgsman et al., 2019), which are also called NKT-like cells. This group of cells possess both cytotoxic capabilities and regulatory function of the immune response via the secretion of pro- or anti-inflammatory cytokines upon activation (Krijgsman et al., 2018). Our study showed no significant change in NK cells but a reduced level of NKT-like cells after IL-17A inhibition. Further studies involving cytokines secretion were needed to confirm the findings.

The current study had some limitations which should be carefully considered. First, AS has different phenotypes regarding axial and peripheral involvement and different HLA-B27 status. It remains questionable how various clinical manifestations are related to immune cell imbalance. Second, longitudinal data with prolonged secukinumab treatment would be helpful to provide more knowledge about the long-lasting alterations of these immune cells. Third, the peripheral proportion of immune cell subsets could partially reflect of how immune system works after the use of IL17A inhibitor. A subsequent function investigation would better explain relevant mechanisms.

## Conclusion

Our prospective study confirmed AS patients had significant alteration of immune cell frequency. Anti-IL-17A therapy (Secukinumab) could reduce inflammation and reverse partial immune cell imbalance. The baseline level of total T cells might be an indicator of good response to secukinumab.

## Data Availability

The raw data supporting the conclusion of this article will be made available by the authors, without undue reservation.
