# Supplementary figures and images for: Dynamics of Adaptive Immune Cell and NK Cell Subsets in Patients With Ankylosing Spondylitis After IL-17A Inhibition by Secukinumab
Source: Front Pharmacol. 2021 Oct 14;12:738316. doi: 10.3389/fphar.2021.738316 (PMC8551761; doi:10.3389/fphar.2021.738316)

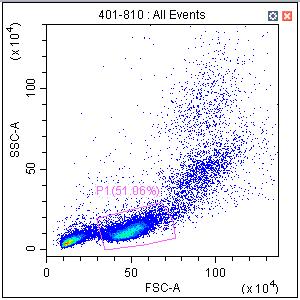

Supplement: Supplementary file 1 [file DataSheet2.zip › raw data/gating/B/1.jpg]

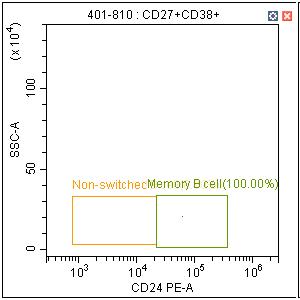

Supplement: Supplementary file 1 [file DataSheet2.zip › raw data/gating/B/10.jpg]

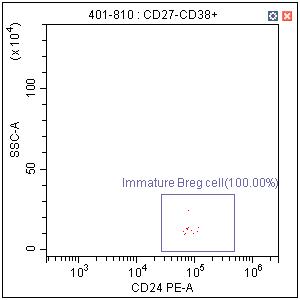

Supplement: Supplementary file 1 [file DataSheet2.zip › raw data/gating/B/11.jpg]

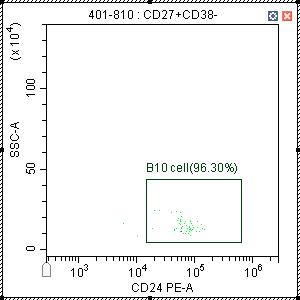

Supplement: Supplementary file 1 [file DataSheet2.zip › raw data/gating/B/12.jpg]

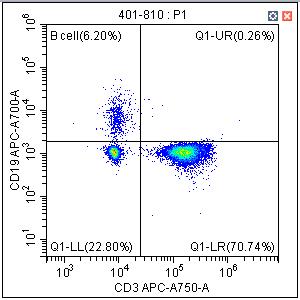

Supplement: Supplementary file 1 [file DataSheet2.zip › raw data/gating/B/2.jpg]

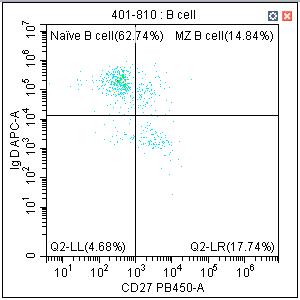

Supplement: Supplementary file 1 [file DataSheet2.zip › raw data/gating/B/3.jpg]

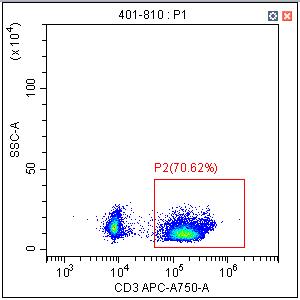

Supplement: Supplementary file 1 [file DataSheet2.zip › raw data/gating/B/4.jpg]

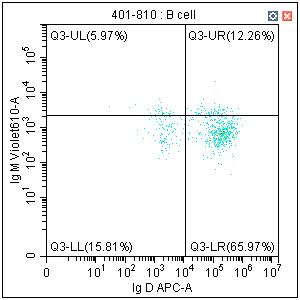

Supplement: Supplementary file 1 [file DataSheet2.zip › raw data/gating/B/5.jpg]

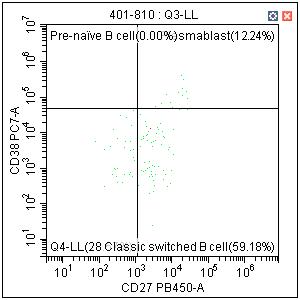

Supplement: Supplementary file 1 [file DataSheet2.zip › raw data/gating/B/6.jpg]

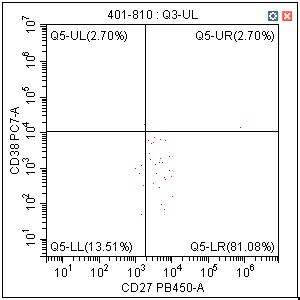

Supplement: Supplementary file 1 [file DataSheet2.zip › raw data/gating/B/7.jpg]

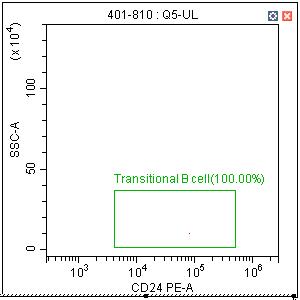

Supplement: Supplementary file 1 [file DataSheet2.zip › raw data/gating/B/8.jpg]

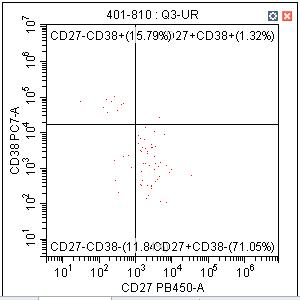

Supplement: Supplementary file 1 [file DataSheet2.zip › raw data/gating/B/9.jpg]

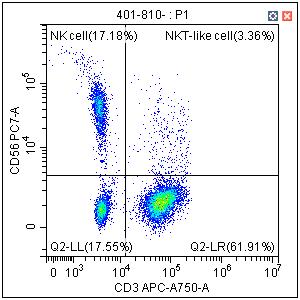

Supplement: Supplementary file 1 [file DataSheet2.zip › raw data/gating/NK and NKT-like cell.jpg]

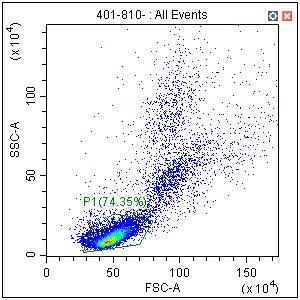

Supplement: Supplementary file 1 [file DataSheet2.zip › raw data/gating/T/1.jpg]

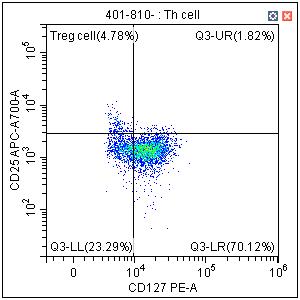

Supplement: Supplementary file 1 [file DataSheet2.zip › raw data/gating/T/10.jpg]

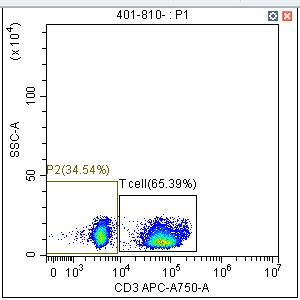

Supplement: Supplementary file 1 [file DataSheet2.zip › raw data/gating/T/2.jpg]

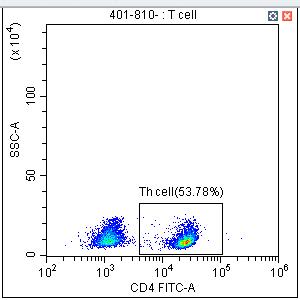

Supplement: Supplementary file 1 [file DataSheet2.zip › raw data/gating/T/3.jpg]

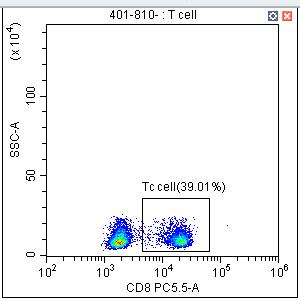

Supplement: Supplementary file 1 [file DataSheet2.zip › raw data/gating/T/4.jpg]

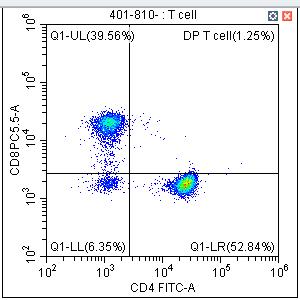

Supplement: Supplementary file 1 [file DataSheet2.zip › raw data/gating/T/5.jpg]

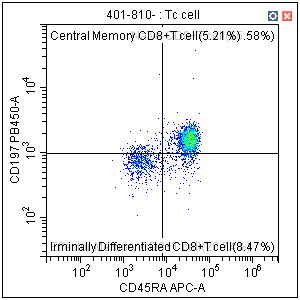

Supplement: Supplementary file 1 [file DataSheet2.zip › raw data/gating/T/6.jpg]

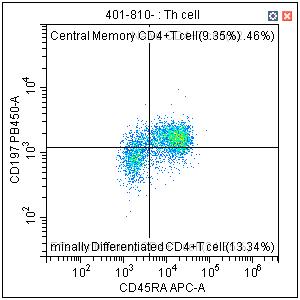

Supplement: Supplementary file 1 [file DataSheet2.zip › raw data/gating/T/7.jpg]

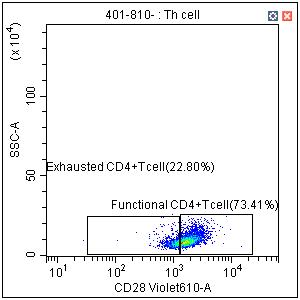

Supplement: Supplementary file 1 [file DataSheet2.zip › raw data/gating/T/8.jpg]

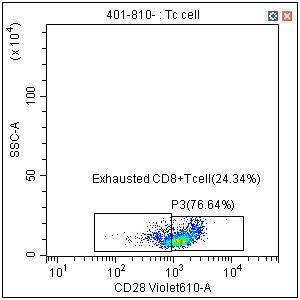

Supplement: Supplementary file 1 [file DataSheet2.zip › raw data/gating/T/9.jpg]

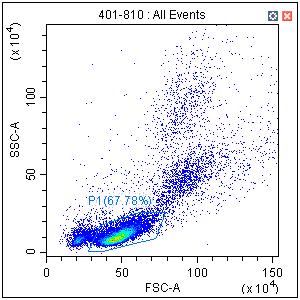

Supplement: Supplementary file 1 [file DataSheet2.zip › raw data/gating/Th/1.jpg]

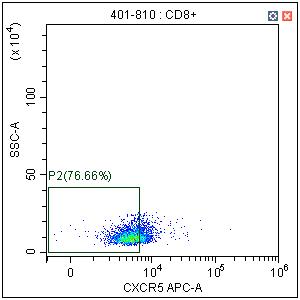

Supplement: Supplementary file 1 [file DataSheet2.zip › raw data/gating/Th/10.jpg]

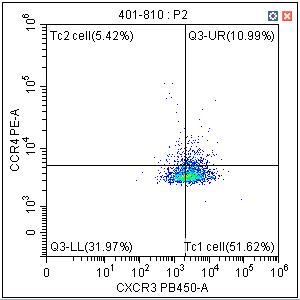

Supplement: Supplementary file 1 [file DataSheet2.zip › raw data/gating/Th/11.jpg]

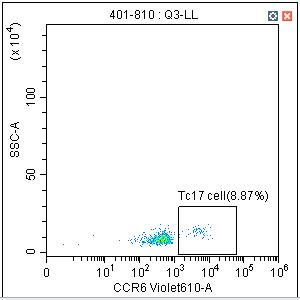

Supplement: Supplementary file 1 [file DataSheet2.zip › raw data/gating/Th/12.jpg]

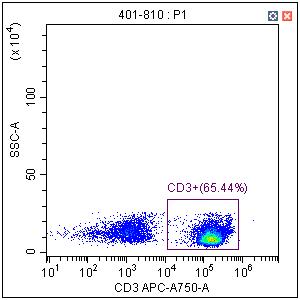

Supplement: Supplementary file 1 [file DataSheet2.zip › raw data/gating/Th/2.jpg]

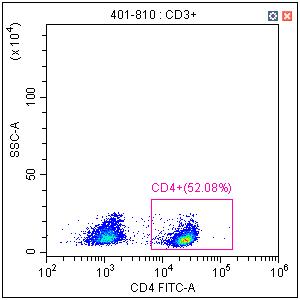

Supplement: Supplementary file 1 [file DataSheet2.zip › raw data/gating/Th/3.jpg]

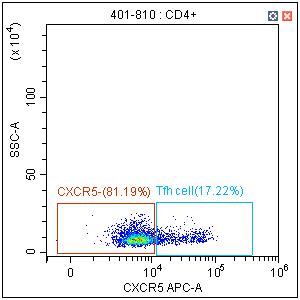

Supplement: Supplementary file 1 [file DataSheet2.zip › raw data/gating/Th/4.jpg]

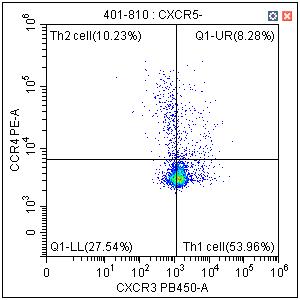

Supplement: Supplementary file 1 [file DataSheet2.zip › raw data/gating/Th/5.jpg]

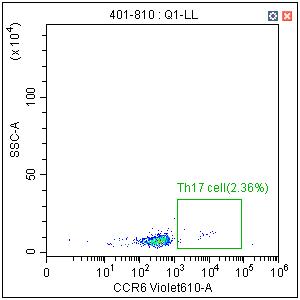

Supplement: Supplementary file 1 [file DataSheet2.zip › raw data/gating/Th/6.jpg]

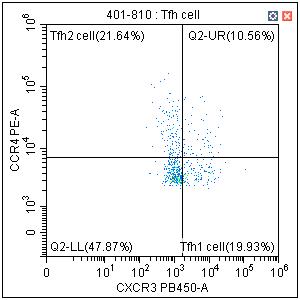

Supplement: Supplementary file 1 [file DataSheet2.zip › raw data/gating/Th/7.jpg]

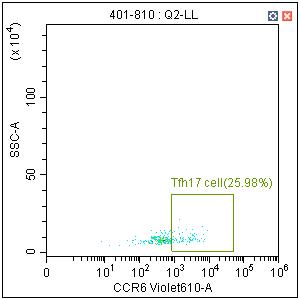

Supplement: Supplementary file 1 [file DataSheet2.zip › raw data/gating/Th/8.jpg]

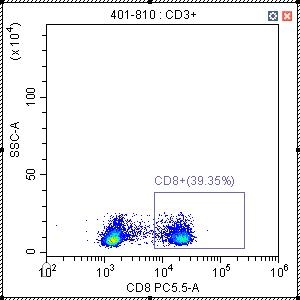

Supplement: Supplementary file 1 [file DataSheet2.zip › raw data/gating/Th/9.jpg]
