# Supplementary material for: Dynamics of Adaptive Immune Cell and NK Cell Subsets in Patients With Ankylosing Spondylitis After IL-17A Inhibition by Secukinumab
Source: Front Pharmacol. 2021 Oct 14;12:738316. doi: 10.3389/fphar.2021.738316 (PMC8551761; doi:10.3389/fphar.2021.738316)
Supplement: Supplementary file 2 [file DataSheet1.docx]

**Supplementary Table 1 The surface markers of lymphocyte and NK cell subsets in the study**

| Lymphocyte subsets | Phenotype |
| --- | --- |
| T cell | CD3+ |
| Th cell | CD3+CD4+ |
| Tc cell | CD3+CD8+ |
| DP T cell | CD3+CD4+CD8+ |
| Naïve CD4+T cell | CD3+CD4+CD45RA+CCR7+ |
| Terminally Differentiated CD4+T cell | CD3+CD4+CD45RA+CCR7- |
| Central Memory CD4+T cell | CD3+CD4+CD45RA-CCR7+ |
| Effector Memory CD4+T cell | CD3+CD4+CD45RA-CCR7- |
| Exhausted CD4+T cell | CD3+CD4+CD28- |
| Functional CD4+T cell | CD3+CD4+CD28+ |
| Treg cell | CD3+CD4+CD25+CD127- |
| Naïve CD8+T cell | CD3+CD8+CCR7+CD45RA+ |
| Terminally Differentiated CD8+T cell | CD3+CD8+CCR7-CD45RA+ |
| Central Memory CD8+T cell | CD3+CD8+CCR7+CD45RA- |
| Effector Memory CD8+T cell | CD3+CD8+CCR7-CD45RA- |
| Exhausted CD8+T cell | CD3+CD8+CD28- |
| Tfh cell | CD3+CD4+CXCR5+ |
| Th1 cell | CD3+CD4+CXCR5-CXCR3+CCR4- |
| Th2 cell | CD3+CD4+CXCR5-CXCR3-CCR4+ |
| Th17 cell | CD3+CD4+CXCR5-CXCR3-CCR4-CCR6+ |
| Tfh1 cell | CD3+CD4+CXCR5+CXCR3+CCR4- |
| Tfh2 cell | CD3+CD4+CXCR5+CXCR3-CCR4+ |
| Tfh17 cell | CD3+CD4+CXCR5+CXCR3-CCR4-CCR6+ |
| Tc1 cell | CD3+CD8+CXCR5-CXCR3+CCR4- |
| Tc2 cell | CD3+CD8+CXCR5-CXCR3-CCR4+ |
| Tc17 cell | CD3+CD8+CXCR5-CXCR3-CCR4-CCR6+ |
| B cell | CD3-CD19+ |
| Naïve B cell | CD3-CD19+CD27-IgD+ |
| MZ B cell | CD3-CD19+CD27+IgD+ |
| Pre-naïve B cell | CD3-CD19+IgM-IgD-CD27-CD38+ |
| Plasma cell | CD3-CD19+IgD-IgM-CD27+CD38+ |
| Classic switched B cell | CD3-CD19+IgD-IgM-CD27+CD38- |
| B10 cell | CD3-CD19+IgD+IgM+CD27+CD38-CD24+ |
| Memory B cell | CD3-CD19+IgD+IgM+CD27+CD38+CD24+ |
| Non-switched B cell | CD3-CD19+IgD+IgM+CD27+CD38+CD24- |
| Immature Breg cell | CD3-CD19+IgD+IgM+CD27-CD38+CD24+ |
| Transitional B cell | CD3-CD19+IgM+IgD-CD27-CD38+CD24+ |
| NK cell | CD3-CD56+ |
| NKT-like cell | CD3+CD56+ |

Th cell, helper T cell; Tc cell, cytotoxic T cell; DP T cell, double-positive T cell; Treg cell, regulatory T cell; Tfh cell, follicular helper T cell; MZ B cell, marginal zone B cell; Breg cell, regulatory B cell; NK cell, nature killer cell.

**Supplementary Table 2 Clinical variables of AS patients and HCs at baseline and after secukinumab treatment**

| **Parameters** | **AS (n=43)** | | **HC (n=47)** | ***p^1^*** | ***p^2^*** |
| --- | --- | --- | --- | --- | --- |
| **Age (years)** | 28.3±9.9 | | 30.6±5.1 | 0.196 | - |
| **Male (n, %)** | 34 (79.1%) | | 31 (66.0%) | 0.165 | - |
| **HLA-B27 positivity (n, %)** | 39 (90.6%) | | - | - | - |
| **Disease duration (years)** | 6.5 (3.1-9.6) | | - | - | - |
|  | Pre-treatment | Post-treatment |  |  |  |
| **CRP (mg/L)** | 17.7 (1.5-23.8) | 2.2 (1.0-6.8) | - | - | <0.001 |
| **ASDAS** | 2.97±1.02 | 1.37±1.04 | - | - | <0.001 |
| **Naïve to TNFi (n, %)** | 24 (55.8%) | | - | - | - |
| **Secukinumab responder (n, %)** | 33 (76.7%) | |  |  |  |
| **Secukinumab nonresponder (n, %)** | 10 (23.3%) | |  |  |  |

Data with normal distribution were represented using mean± SD, and those with non-normal distribution were presented by median (interquartile range). ^1^ Comparison of immune cells using the independent t tests between AS patients and HC at baseline; ^2^ Comparison of immune cells using the paired Wilcoxon tests and paired t tests in AS patients before and after treatment with secukinumab. AS, ankylosing spondylitis; HC, healthy control; CRP, C-reactive protein; ASDAS: Ankylosing Spondylitis Disease Activity Score; TNFi, tumor necrosis factor inhibitor.

**Supplementary Table 3 Comparisons of immune cell frequency in patients pre- and post- treatment and HC**

|  | Baseline | After Treatment | HC | *p*^1^ | *p*^2^ |
| --- | --- | --- | --- | --- | --- |
| T-cell (%) | 55.0 (42.4-64.7) | 43.9 (33.1-50.1) | 72.5 (63.6-76.1) | <0.0001^****^ | <0.0001^****^ |
| Th | 51.1 (42.5-58.7) | 43.7 (37.6-50.7) | 57.5 (44.3-63.2) | 0.001^***^ | 0.074 |
| Tc | 41.6±11.9 | 41.1±11.9 | 30.3±7.8 | 0.764 | <0.0001^****^ |
| CD4/CD8 | 1.3±0.6 | 1.1±0.6 | 1.9±0.7 | 0.017^*^ | <0.0001^****^ |
| DP T | 0.7 (0.4-1.2) | 0.6 (0.4-1.2) | 1.1 (1.3-2.3) | 0.108 | 0.005^**^ |
| CD4+ T-cell subtype (%) | | | | | |
| Naïve CD4+ T | 44.4±18.2 | 49.2±15.0 | 22.2±11.0 | 0.137 | <0.0001^****^ |
| TD CD4+ T | 3.6 (2.0-12.2) | 2.4 (1.3-3.9) | 24.0 (15.3-30.8) | 0.019^*^ | <0.0001^****^ |
| CM CD4+ T | 16.7±8.9 | 22.5±10.1 | 7.9±3.5 | 0.004^**^ | <0.0001^****^ |
| EM CD4+ T | 27.5±11.9 | 22.6±13.2 | 45.7±9.4 | 0.023^*^ | <0.0001^****^ |
| Exhausted CD4+ T | 7.6 (4.5-17.8) | 7.0 (2.9-14.3) | 1.6 (0.5-4.4) | 0.604 | <0.0001^****^ |
| Functional CD4+ T | 92.4 (81.2-95.7) | 91.4 (84.0-96.3) | 98.4 (95.6-99.6) | 0.894 | <0.0001^****^ |
| Treg | 3.5 (2.3-4.5) | 2.5 (1.6-3.0) | 3.5 (2.9-7.8) | <0.0001^****^ | 0.187 |
| CD8+ T-cell subtype (%) | | | | | |
| Naïve CD8+ T | 41.6±19.4 | 42.3±19.4 | 22.8±14.2 | 0.767 | <0.0001^****^ |
| TD CD8+ T | 18.4 (12.1-33.8) | 15.7 (9.2-28.7) | 46.8 (41.6-62.5) | 0.091 | <0.0001^****^ |
| CM CD8+ T | 2.4 (1.4-4.5) | 3.2 (1.7-6.1) | 0.6 (0.4-0.9) | 0.091 | <0.0001^****^ |
| EM CD8+ T | 32.7±12.9 | 31.8±13.1 | 27.3±10.1 | 0.641 | 0.027^*^ |
| Exhausted CD8+ T | 52.2±25.7 | 36.7±19.5 | 22.5±20.0 | <0.0001^****^ | <0.0001^****^ |
| Th1 | 11.4 (7.0-21.5) | 18.1 (10.0-66.9) | 17.1 (12.7-22.5) | 0.003^**^ | 0.046^*^ |
| Th2 | 10.3 (7.3-18.1) | 10.1 (6.3-14.0) | 12.4 (8.6-15.2) | 0.134 | 0.292 |
| Th17 | 1.5 (0.8-3.9) | 1.7 (0.7-4.4) | 1.1 (0.6-1.8) | 0.579 | 0.017^*^ |
| Tfh | 18.0 (11.7-27.0) | 28.1 (16.7-69.5) | 18.7 (15.7-21.1) | <0.0001^****^ | 0.84 |
| Tfh1 | 13.9 (10.1-19.0) | 20.1 (14.1-29.4) | 13.9 (11.7-18.4) | 0.024^*^ | 0.812 |
| Tfh2 | 29.4 (23.3-36.3) | 16.3 (11.2-22.6) | 34.8 (27.3-39.0) | <0.0001^****^ | 0.062 |
| Tfh17 | 7.9 (3.7-11.8) | 6.7 (3.3-10.7) | 5.0 (3.3-6.8) | 0.866 | 0.013^*^ |
| Tc1 | 18.8 (8.7-32.9) | 42.3 (26.2-55.1) | 54.6 (47.5-65.1) | <0.0001^****^ | <0.0001^****^ |
| Tc2 | 9.9 (4.9-14.8) | 4.3 (2.7-12.5) | 2.0 (1.2-3.3) | 0.037^*^ | <0.0001^****^ |
| Tc17 | 1.5 (0.4-2.4) | 2.6 (0.8-4.2) | 4.2 (1.8-9.1) | 0.158 | <0.0001^****^ |
| Th17/Treg | 0.4 (0.3-1.6) | 0.7 (0.3-1.6) | 0.3 (0.07-0.6) | 0.837 | 0.003^**^ |
| B cell subtype (%) | 6.4 (3.9-14.0) | 4.6 (2.7-6.8) | 9.4 (6.4-12.4) | 0.0013^**^ | 0.194 |
| Naïve B | 55.9 (36.6-65.8) | 67.5 (54.5-78.7) | 58.7 (44.4-69.9) | 0.0052^**^ | 0.394 |
| MZ B | 10.2±11.5 | 9.3±5.9 | 9.2±6.5 | 0.586 | 0.602 |
| Pre-naïve B | 1.5 (0.6-2.2) | 0.2 (0.0-1.3) | 4.7 (2.0-18.0) | 0.002^**^ | <0.0001^****^ |
| Plasma cell | 3.6 (0.1-5.8) | 2.2 (0.5-4.4) | 7.0 (2.9-10.6) | 0.378 | 0.001^***^ |
| Classic switched B | 4.0 (1.7-6.1) | 4.6 (2.4-8.3) | 5.6 (3.3-8.0) | 0.31 | 0.036^*^ |
| B10 | 2.3 (0.7-5.2) | 5.5 (1.6-9.1) | 0.5 (0.3-1.3) | 0.009 | <0.0001^****^ |
| Memory B | 6.7 (3.6-39.5) | 1.2 (0.4-3.4) | 0.8 (0.4-1.4) | <0.0001^****^ | <0.0001^****^ |
| Non-switched B | 7.2 (3.3-12.6) | 0.0 (0.0-0.4) | 0.1 (0.04-0.2) | <0.0001^****^ | <0.0001^****^ |
| Immature Breg | 0.0 (0.0-0.4) | 2.7 (0.0-8.3) | 0.4 (0.1-0.7) | <0.0001^****^ | <0.0001^****^ |
| Transitional B | 0.08 (0.0-0.9) | 0.1 (0.0-0.4) | 0.03 (0.0-0.1) | 0.67 | 0.05 |
| NK | 4.8 (1.9-7.8) | 8.5 (4.6-15.5) | - | 0.116 | - |
| NKT-like | 10.0 (5.2-13.4) | 6.0 (3.4-10.0) | - | 0.047^*^ | - |

Data with normal distribution were represented using mean± SD, and those with non-normal distribution were presented by median (interquartile range). Th, helper T; Tc, cytotoxic T; DP, double positive; TD, terminally differentiated; CM, central memory; EM, effector memory; Treg, regulatory T; Tfh, follicular helper T; MZ B, marginal zone B; Breg, regulatory B cell; NK, nature killer. ^1^ Comparison of immune cells using the independent t tests or the Mann-Whitney U tests between AS patients and HCs at baseline; ^2^ Comparison of immune cells using the paired Wilcoxon tests or paired t tests in AS patients before and after treatment with secukinumab.

^*^*, p< 0.05*; ^**^*, p< 0.01*; ^***^*, p≤ 0.001*；^****^*, p< 0.0001.*
