# Supplementary material for: Dynamics of Adaptive Immune Cell and NK Cell Subsets in Patients With Ankylosing Spondylitis After IL-17A Inhibition by Secukinumab
Source: Front Pharmacol. 2021 Oct 14;12:738316. doi: 10.3389/fphar.2021.738316 (PMC8551761; doi:10.3389/fphar.2021.738316)
Supplement: Supplementary file 3 [file Image1.pdf]

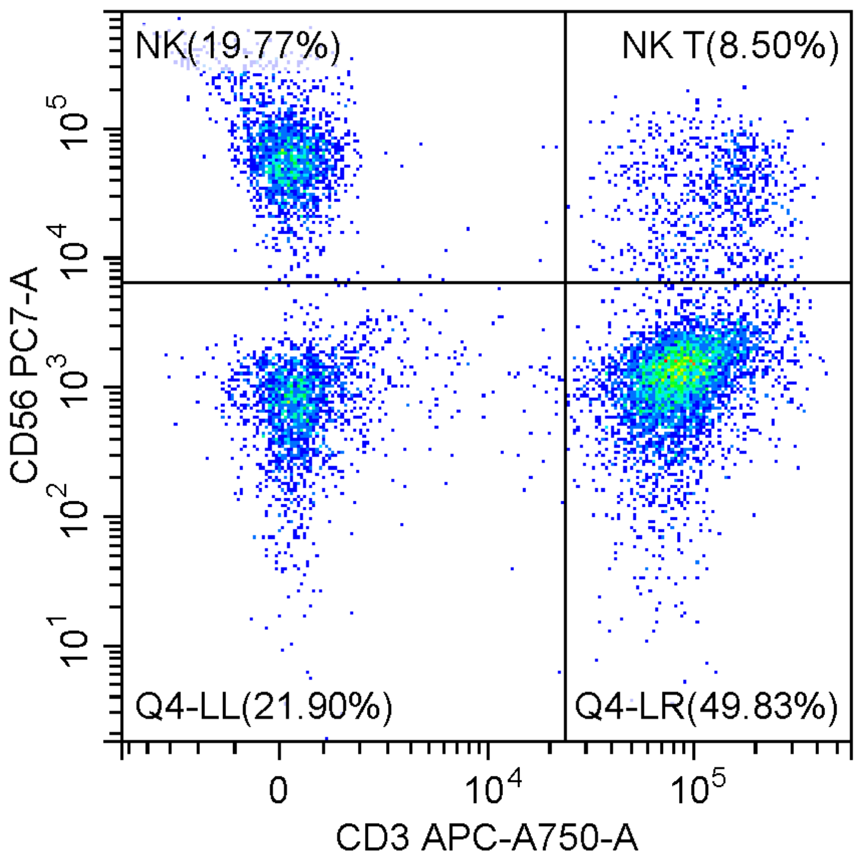

Supplementary Figure 1 Representative gating strategy to identify NK and NKT-like cells. NK, nature kill.
